# Innovative strategies on accelerating skin wound healing in dogs using chitosan-coated zinc oxide nanoparticles delivered by two different routes

**DOI:** 10.2478/jvetres-2026-0050

**Published:** 2026-09-16

**Authors:** Marwa H. Hamdy, Eman I. Hassanen, Marwa A. Ibrahim, Neven H. Hassan, Sherif H. Elmosalamy, Khaled Y. Farroh, Mohamed M. Bahr

**Affiliations:** 1 Department of Veterinary Clinical Sciences, Faculty of Veterinary Medicine, Jordan University of Science and Technology, Irbid 22110, Jordan; 2 Department of Pathology, Department of Biochemistry and Molecular Biology, Department of Physiology, Department of Surgery, Faculty of Veterinary Medicine, Cairo University, Giza 12613, Egypt; 3 Department of Biochemistry and Molecular Biology, Department of Physiology, Department of Surgery, Faculty of Veterinary Medicine, Cairo University, Giza 12613, Egypt marwaibrahim@cu.edu.eg; 4 Department of Physiology, Department of Surgery, Faculty of Veterinary Medicine, Cairo University, Giza 12613, Egypt; 5 Nanotechnology and Advanced Materials Central Lab., Regional Center for Food and Feed, Agricultural Research Center, Giza 12619, Egypt; 6 Regional Center for Food and Feed, Agricultural Research Center, Giza 12619, Egypt; 7 Department of Surgery, Faculty of Veterinary Medicine, Cairo University, Giza 12613, Egypt

**Keywords:** angiogenesis, gene expression, nanomedicine, oxidative stress, wound repair

## Abstract

**Introduction:**

Zinc-oxide nanoparticles (ZnO NPs) have shown promise for their antibacterial, anti-inflammatory and regenerative capabilities. Chitosan-coated metal-oxide NPs have higher solubility and bioactivity than uncoated ones, making them an effective biomaterial for medical uses. This study aimed to exploit the complimentary features of chitosan-coated ZnO NPs (CS/ZnO NPs) to overcome existing limits in wound care treatments. It delivered the nanoparticles by two key routes: subcutaneous (s/c) injection and topical (T) application.

**Material and Methods:**

Fifteen dogs were divided into three groups (n = 5) as follows: a control group, a group treated with s/c injection of CS/ZnO NPs (25 ppm) once a week for two weeks and a group treated by daily T application of CS/ZnO NPs (25 ppm) for two weeks. Wounds were monitored grossly and microscopically at 0, 3, 7, 14 and 21 d post treatment. Blood was drawn before the first and after the last treatment for liver and kidney function biomarker and blood morphology evaluation.

**Results:**

Administration of CS/ZnO NPs either s/c or T accelerated wound closure without affecting liver or kidney function, but the s/c route caused a significant deterioration in HGB, HCT, RBC, WBC and PLT counts compared with the T route. Microscopic examination revealed improved epithelialisation, decreased inflammatory cell infiltration, greater collagen deposition and superior tissue organisation in wounds treated with CS/ZnO NPs over time, confirmed by increasing vascular endothelial growth factor and α-smooth muscle actin immunoexpression. The nanoparticles also boosted the transcript levels of the *VEGFA* (vascular endothelial growth factor A) and *TNFα* genes at all time points.

**Conclusion:**

This study demonstrates the benefits of nanotechnology in wound care and emphasises the need for additional research to enhance nanoparticle formulations for therapeutic applications.

## Introduction

Wound healing is a complicated and extended biological process that requires the careful coordination of different cells and molecules. This process is essential for keeping the body intact and protecting it from harm. The stages of wound healing include haemostasis, inflammation, proliferation and remodelling, with each stage being important for repairing and rebuilding tissue (9). Even with improvements in wound care, long-lasting wounds, infections and slow healing still present major health issues globally, making it necessary to create new treatment methods.

In recent years, nanotechnology has become a promising area in which to develop wound healing improvement materials, providing new ways to solve problems related to traditional wound care methods (3). The use of nanomaterials in wound healing has gained a lot of attention because of their special properties, which can help promote tissue growth, fight infections and improve the healing environment. Zinc oxide nanoparticles (ZnO NPs) are some of these nanomaterials. These have strong antimicrobial, antioxidant and anti-inflammatory effects, all of which are important for successful wound healing (3). The antimicrobial activity of zinc oxide nanoparticles helps prevent infections by stopping the growth of many types of harmful pathogens, such as bacteria, fungi and viruses. Oxidative stress harms wound healing by damaging cells and prolonging the inflammation stage. By removing reactive oxygen species (ROS), ZnO NPs help create a better environment for tissue repair and growth. Their anti-inflammatory effects are also important for wound healing. Although inflammation is a necessary part of the initial healing process, excessive or long-lasting inflammation can slow down healing and cause wounds to become chronic. Zinc oxide nanoparticles contribute to managing inflammation, possibly ensuring a balance between the needed inflammation and the progress to later healing stages (22).

However, ZnO NPs alone may have some limitations on their effectiveness, such as poor bioavailability and their burst release when gradual release over time is desired. To overcome these issues, researchers have looked at natural biopolymers as potential carriers for these nanoparticles. Chitosan (CS), a natural biopolymer made from chitin, has been widely studied for its own healing properties and its ability to deliver drugs effectively (11). Chitosan has many qualities that make it a great choice for wound healing. Its biocompatibility ensures it causes little to no negative reaction when applied to wounds, and its biodegradability allows it to break down and be removed from the body over time. Chitosan also has haemostatic properties, staunching bleeding in fresh wounds (11). Additionally, its positive charge boosts its antimicrobial effects. The combination of CS and ZnO NPs possibly improves different parts of the healing process. Chitosan can enhance the stability and availability of ZnO NPs while also augmenting healing through its own properties. The benefits of CS-coated zinc-oxide nanocomposites (CS/ZnO NPs) support the healing of chronic wounds as well as acute wounds; acting to counter chronic wounds’ frequent ongoing inflammation and mitigating the consequent poor healing (6).

This paper aims to look closely at how CS/ZnO NPs affect wound healing in dogs. It focuses on important parts of the healing process, such as controlling inflammation, facilitating tissue regeneration and increasing wound contraction, and it determines overall healing results. Additionally, a comparison is provided between a topical dressing and subcutaneous injection regarding the wound healing activity of CS/ZnO NPs in dogs.

## Material and Methods

### Preparation of chitosan-coated zinc oxide nanoparticles

Nanoparticles were produced using the precipitation procedure. Typically, 7.1883 g of zinc sulphate heptahydrate (ZnSO_4_ 7H_2_O, 99% purity; Sigma-Aldrich, St. Louis, MO, USA) was dissolved in 50 mL of deionised water (Milli-Q; Merck Millipore, Billerica, MA, USA) using a magnetic stirrer. Then, dropwise additions of sodium hydroxide (50 mL, 1 M) (98% purity, Sigma-Aldrich) were made under continuous stirring for a further 30 min. The precipitates were filtered and washed multiple times with deionised water. After that, the precipitates were dried for 24 h at 60°C and calcined for 2 h at 500°C (18).

Chitosan-coated ZnO NPs were prepared according to Zabihi *et al*. (34) with some modifications. Briefly, CS with molecular weight of 100–300 kDa and viscosity of 50 to 200mPa·s (ORISON, Tianjin, China) was prepared in an aqueous solution (0.2% w/v) by dissolving it in acetic acid solution (1% v/v, 99–100%; Riedel-de Haën, Seelze, Germany) at room temperature. Subsequently, tripolyphosphate (TPP) (technical grade, 85%; Sigma-Aldrich) as a cross-linking agent was dissolved in 10 mL of deionised water to a final concentration of 0.6 mg/mL. The TPP solution was then added to the CS solution dropwise (0.3 mL/min) under vigorous magnetic stirring for 30 min. Zinc-oxide NPs (0.1% w/v) were added to the CS solution under probe sonication in a UP400ST ultrasonicator (Hielscher, Teltow, Germany) for 5 min at 50% amplitude in an ice bath to avoid overheating in the presence of Tween 80 (Sigma-Aldrich) as a surfactant to reduce the nanoparticles’ hydrodynamic diameter. The suspension of CS/ZnO NPs was freeze-dried before further use or analysis (34).

### Characterisation of the prepared nanoparticles

The morphology of the prepared CS/ZnO NPs was imaged with a high-resolution transmission electron microscope operating at an accelerating voltage of 200 kV (Tecnai G2; FEI, Eindhoven, the Netherlands). The diluted solution was ultra-sonicated for 5 min to reduce particle aggregation. Using a micropipette, three drops from the sonicated solution were deposited on a carbon-coated copper grid and left to dry at room temperature. Images were captured for morphological evaluation. The chemical structure of the prepared NPs was assessed using X-ray diffraction (XRD) in an X’pert PRO diffractometer (PANalytical, Almelo, the Netherlands). The corresponding XRD pattern was recorded in the scanning mode using a Cu-anode X-ray tube (Kα radiation, λ = 1.54 Å) at 40 kV and 30 mA. The obtained diffraction pattern was interpreted by the standard ICDD library installed in PDF4 software.

### Ethical approval

All procedures were carried out in compliance with the UK Guidance on the Operation of the Animals (Scientific Procedures) Act 1986 and the ARRIVE 2.0 guidelines (26). The experiment was approved by Cairo University Institutional Animal Care Committee under No. CU/II/F/8/24. All dog owners were advised that their animals would be used for research; had the purpose, procedures and potential risks and benefits explained to them; and gave written informed consent for their animals’ use.

### Animals

Fifteen apparently healthy male mongrel dogs were enrolled. They were aged 1–2 years with average body weight of 15–20 kg. The dogs were kept in separate kennels at the Department of Surgery, Anaesthesiology and Radiology, Faculty of Veterinary Medicine, Cairo University. They were granted unrestricted access to regular food and water. They were acclimatised for two weeks prior to the experiment to guarantee their health. During the acclimatisation period, a physical examination was performed on all dogs, and blood samples were collected from the jugular vein to perform liver and kidney function tests to ensure their healthy status. All dogs used during the experiment received proper treatment and were returned in good health at the end of the study.

### Induction of skin wound

After the two-week acclimatisation period, the experimental dogs were fasted overnight, then given a general anaesthetic regimen as follows: premedication with atropine sulphate (0.1 mg/kg body weight) *via* subcutaneous injection, xylazine (1 mg/kg body weight *via* intramuscular injection and ketamine (10 mg/kg body weight) *via* intravenous injection. Using a dermal punch, bilateral full-thickness circular skin wounds of 3-cm diameter were made on each dog’s back in an aseptic environment (16).

### Treatment regimen

After 24 h of wound induction, the 15 dogs were randomly divided into three equal groups. In Group I (control), wounds on the right side were treated with topical hydrogel, while wounds on the left side underwent s/c infiltration with 0.9% normal saline. In Group II, both wounds were treated with s/c injections of CS/ZnO NPs (25 ppm) once a week for two weeks. In Group III, both wounds were treated with daily topical application of CS/ZnO NPs (25 ppm) mixed with hydrogel for 14 d.

Wounds in all groups were covered with dressing bandages, and all dogs received a systemic pain killer and a broad-spectrum antibiotic for 3 d consecutively. The wounds were photographed at 0, 3, 7, 14 and 21 d post-treatment (PT) to calculate wound-size reduction percentage using the following formula:

$$\text{wound-size reduction }=\left({\text{A}}_{\text{i}}-{\text{A}}_{\text{f}}\right)/{\text{A}}_{\text{i}}\times 100$$

where (A_i_) is the initial wound area and (A_f_) is the wound area at the time point (35).

### Humane endpoints

The undertaking was given that if any enrolled dogs developed an infection or had delayed wound healing during the study, an immediate clinical assessment would be conducted and medical intervention initiated, which would include wound debridement, administering systemic or topical antimicrobial agents and providing supportive therapies as deemed necessary. Affected animals would be removed from the experimental protocol if they did not respond to these interventions or displayed persistent signs of significant pain, distress or a decline in overall health, and then would be provided veterinary care adhering to recognised animal welfare standards (26). These procedures aimed to minimise discomfort, and the undertaking aimed to maintain the highest standards of animal well-being throughout the investigation.

### Sample collection

The dogs were given a general anaesthetic regimen as previously mentioned, and blood samples were collected from the jugular vein at 0 and 21 d PT. Some of these samples were collected in a heparinised tube and used for haematological parameter evaluation, and some were centrifuged to collect serum samples used for biochemical parameter evaluation. Skin wound biopsies were taken at 7, 14 and 21 d PT from the wound periphery. Some of these samples were fixed in 10% neutral-buffered formalin until use for histological and immunohistochemical analyses, and some were frozen at –80°C until use for molecular assays.

### Haematological and biochemical parameters

Haematological analysis (RBC, WBC and PLT counts; HGB; PCV; MCV and MCHC; and a differential leucocyte count using Giemsa stain) was made manually of the collected fresh whole-blood samples as previously described (10). Serum levels of AST, ALT, total protein, ALB, urea and CR were determined using specialised kits from Biodiagnostic Co. (Cairo, Egypt).

### Oxidative stress evaluation

Serum redox status was evaluated by measuring MDA, total antioxidant capacity (TAC) levels, and catalase enzymatic activity (CAT) using the standardised kits purchased from Biodiagnostic Co. (Cairo, Egypt) and following the manufacturer’s instructions.

### Determination of the transcript levels of *TNFα* and *VEGFA* (vascular endothelial growth factor A)

The total RNA was isolated using an RNeasy Kit (Qiagen, Hilden, Germany), then both the quality and quantity of the extracted RNA were assessed using a NanoDrop spectrophotometer (Thermo Fisher Scientific, Waltham, MA, USA). Reverse transcription was carried out using the Thermo Scientific cDNA Synthesis Kit according to the manufacturer’s protocol. The reaction was incubated at 25°C for 10 min and at 42°C for 60 min and then inactivated by heat at 85°C for 5 min. Amplification of the target genes was in SYBR Green PCR Master Mix, and a no-template control was included (Thermo Scientific, Catalog No. 4309155)). The primer sequences of the studied genes are presented in Table 1, as is that of the primer for the *GAPDH* (glyceraldehyde 3-phosphate dehydrogenase) housekeeping gene used to normalise data (23). The reference gene sequences used for primer design and sequence identification were from GenBank, and their accession numbers are also in Table 1. A Bio-Rad Real-Time PCR System (Hercules, CA, USA) was used and set as follows: initial denaturation at 95°C for 3 min; and 40 cycles of denaturation at 95°C for 15 s, annealing at 58°C for 30 s and extension at 72°C for 30 s. The melting curve was analysed at 65–95°C to confirm specific amplification. The relative expression fold change was calculated using the ΔΔCt method (25).

**Table 1.** Primer sequences used for a qRT-PCR to evaluate *TNFα* and *VEGFA* RNA in whole blood from dogs with experimental skin wounds

Gene symbol	Gene description	Accession number	Primer sequence
*TNFα*	Tumour necrosis factor	NM_001003244.4	F: 5′-CTCTCTGCCATCAAGAGCCC-3′ R: 5′-CTAAGCCTGAAGGGGGTGAG-3′
*VEGFA*	Vascular endothelial growth factor A	NM_001003175.2	F: 5′-CCCGGTATAAACCCTGGAGC-3′ R: 5′-ACGCGAGTCTGTGTTTTTGC-3′
*GAPDH*	Glyceraldehyde 3-phosphate dehydrogenase	NM_001003142.2	F: 5′-CGGGAAACTTGTCATCAACGG-3′ R: 5′-TTTGGCTAGAGGAGCCAAGC-3′

F – forward; R – reverse

### Histopathological examination

Formalin-fixed wound samples were processed by traditional procedures using graded alcohol and xylene based on the standard protocol (5). Then all samples were embedded in paraffin wax, sectioned at 4.5 µm and stained with HE. Some sections were stained with Masson’s trichrome (MTC) to visualise collagen fibres. A BX43 light microscope (Olympus, Tokyo, Japan) was used to examine all sections, and a DP27 digital camera (Olympus) linked to cellSens Dimension software (Olympus) was used to capture images of various microscopic fields that were used for further histopathological scoring.

The degree of wound healing across groups was assessed using an ordinal semi-quantitative scoring system (8). Inflammatory cell infiltration, fibroplasia, angiogenesis, collagen deposition and re-epithelisation were the phases of wound healing that were scored blindly in every region. A four-point system was employed to rate the advancement of each healing phase, as follows: – – zero (no changes), + – <25% (slight changes), ++ – 25–50% (moderate changes), +++ – 5075% (marked changes) and ++++ – >75% (extensive changes). The MTC staining intensity was quantified in all experimental groups before and after wound induction using ImageJ software (31). Next, the percentage of collagen stain intensity in each group was calculated according to the following formula:

(mean collagen stain intensity of group/mean collagen stain intensity of normal dermis)×100.

### Immunohistochemistry

Following a microwave retrieval of the antigen, the wound sections were blocked with 1% bovine serum albumin and incubated overnight with primary antibodies against α-smooth muscle actin (aSMA) and vascular endothelial growth factor (VEGF) (Abcam, Cambridge, UK). Then they were incubated using the reagents in an antigen detection system (Power-Stain 1.0 Poly HRP DAB (3,3′-diaminobenzidine tetrahydrochloride) Kit; Sakura Finetek, Tokyo, Japan). Sections were finally counterstained with haematoxylin, mounted using distrene, plasticiser and xylene medium and viewed under a light microscope for further examinations. Image J software was used to calculate the mean percentage of positive immune-reacted area (7, 31).

### Statistical analysis

The amassed data were analysed with SPSS v. 25 software using both one-way and repeated-measures ANOVA (IBM, Armonk, NY, USA). Data were calculated as means ± SEM. Significant difference was determined at P-value ≤ 0.05.

## Results

### Characterisation of the prepared nanoparticles

High-resolution transmission electron microscope images demonstrated nearly spherical NPs with an average size of 16.2 nm (Fig. 1A). The presence of CS and ZnO and the absence of impurity phases is evident from the XRD image (Fig. 1B). The peaks for CS appeared at 2θ value of the broad peak around 16.8–29.9°. The peaks at 2θ which were at 31.97°, 34.47°, 36.44°, 47.72°, 56.98°, 63.04° and 68.32° were attributed to the (100), (002), (101), (102), (110), (103) and (112) crystal planes, indicating that the crystalline structure of synthesised ZnO presented the hexagonal phase structure of zincite (Joint Committee on Powder Diffraction Standards reference pattern 01-075-1526). The results showed that the synthesised nanoparticles were ZnO NPs, because the positions and relative intensities of all the diffraction peaks of the samples were consistent with the crystalline pattern of zinc oxide.

**Fig. 1.**
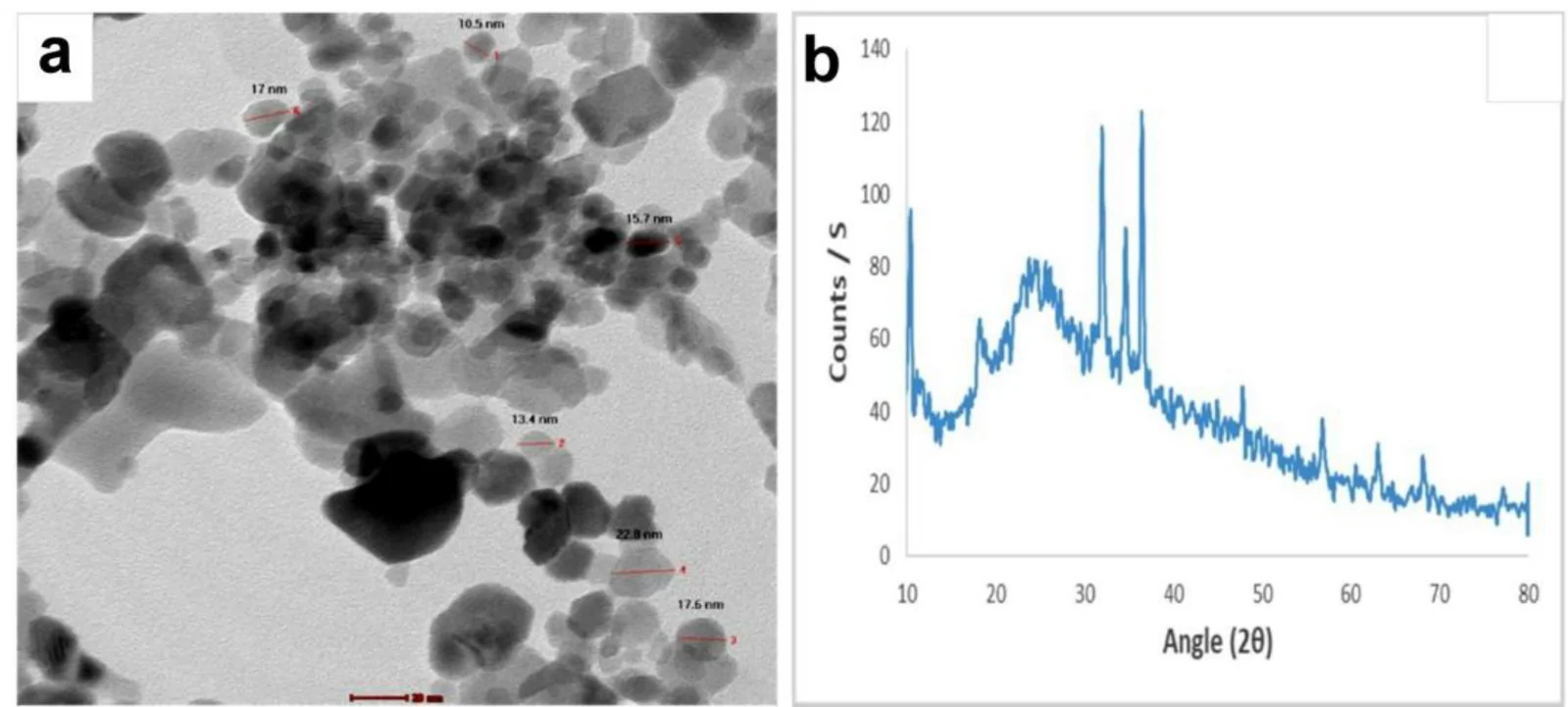
Characterisation of chitosan-coated zinc-oxide nanoparticles (CS/ZnO NPs). a – high-resolution transmission electron microscope image showing nearly spherical particles with an average size of 16.2 nm; b – x-ray diffraction pattern analysis indicating the formation of CS/ZnO NPs

### Macroscopic assessment of wound healing

The skin wounds of the control untreated group did not show any changes in size until the 7^th^ day. Thereafter, they were slightly reduced in size on the 14^th^ day and moderately reduced on the 21^st^ day. Regarding the CS/ZnO NP-treated groups, a remarkable reduction in wound size was observed from day 14 compared with the control untreated group; the wounds had closed by the 21^st^ day PT (Fig. 2).

**Fig. 2.**
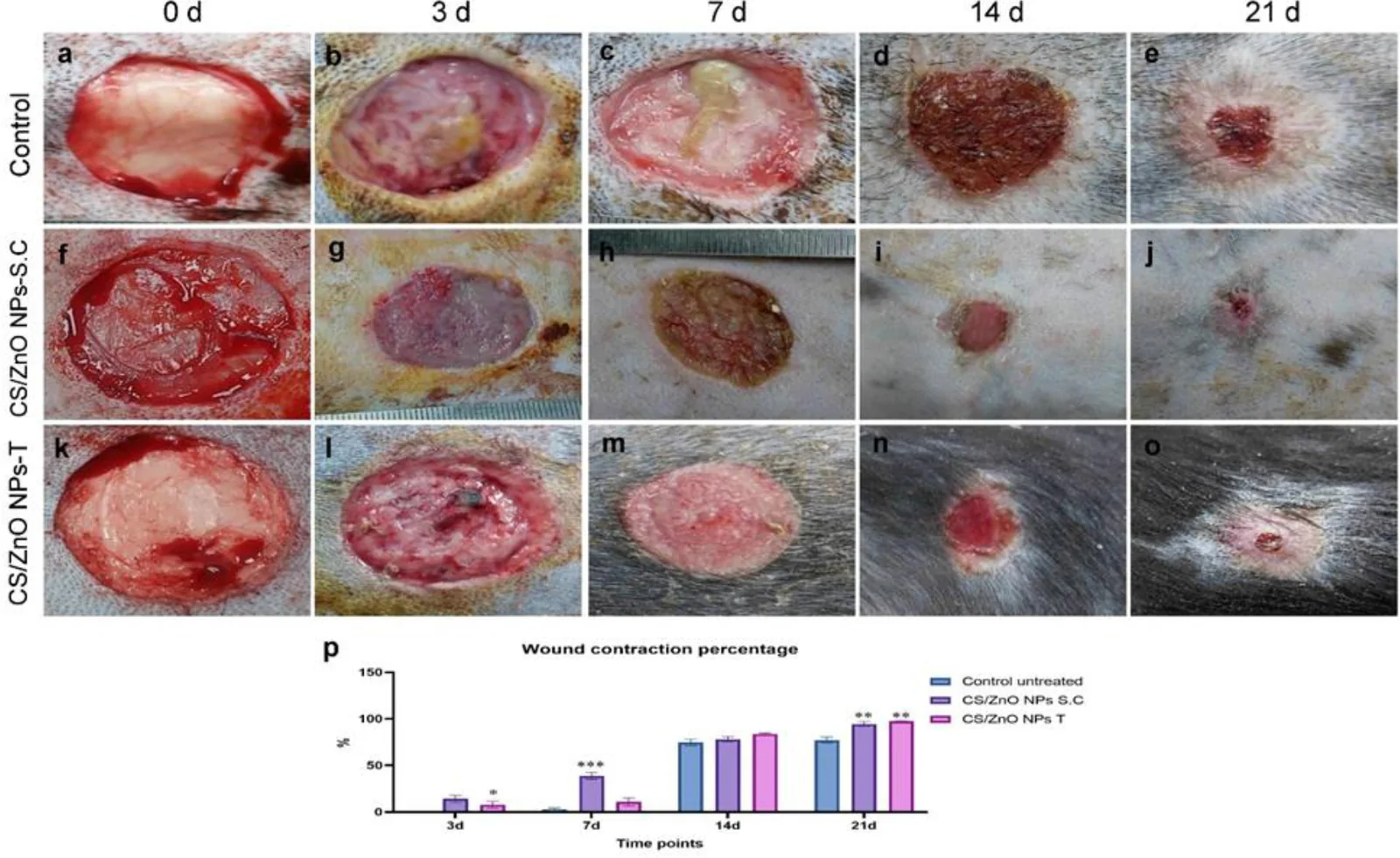
Macroscopic observation of a wound in a dog’s skin after applying or not applying chitosan-coated zinc-oxide nanoparticles (CS/ZnO NPs) on day 0 and the 3^rd^, 7^th^, 14^th^ and 21^st^ days post treatment (PT). a–e – Control untreated group; f–j – CS/ZnO NP subcutaneously (s/c) injected group treated once weekly at 25 ppm; k–o – CS/ZnO NPs topically (T) applied group treated daily at 25 ppm; p – Bar chart representing wound contraction percentages in the treatment groups on the 3^rd^, 7^th^, 14^th^ and 21^st^ days PT. */**/*** – significant difference between groups at the same time-point at P ≤ 0.05/≤ 0.01/≤ 0.001. Values are expressed as means ± SEM

### Wound contraction percentage

There was a significantly higher wound contraction percentage in the CS/ZnO NP-injected group than in the other groups on the 3^rd^ and 7^th^ day PT. There were no significant differences between groups on the 14^th^ day PT; however, there was subsequently, when higher wound contraction percentages were recorded in both CS/ZnO NP-treated groups than in the control group at 21 d PT (Fig. 2p).

### Haematological profile

No significant differences between the groups’ haematological parameters were noted at 0 d (Table 2). A significant reduction of HGB, HCT, RBC count and MCHC with substantial elevations of WBCs and PLT was observed in the untreated group from the 0-d values to the 21-d PT values. The CS/ZnO NP s/c-injected group similarly showed substantial reductions in HGB, HCT and RBC count with significant elevations of WBCs and PLT between the same two time points. However, the CS/ZnO NPs T group’s parameters mostly did not differ significantly, only PLT being significantly elevated at 21 d PT. On the last PT day, the HGB, HCT, RBC count and PLT were significantly higher in the groups receiving CS/ZnO NPs either T or s/c compared to the untreated group, and there was no significant difference between the two NP administration groups. Interestingly, after 21 d the group that received T CS/ZnO NPs had a significantly lower WBC count when compared to the untreated group and the group that received the same treatment s/c.

**Table 2.** Effect on canine haematological parameters on days 0 and 21 of chitosan-coated zinc-oxide nanoparticle (CS/ZnO NP) subcutaneous injection and topical application used in dog skin wound healing

Parameter	Untreated	CS/ZnO NP s/c	CS/ZnO NP T
Day 0 HGB (g/dL)	13.33 ± 0.37 ^A^	12.83 ± 0.24 ^A^	12.63 ± 0.19 ^A^
Day 0 HCT %	35.67 ± 0.58 ^A^	36.00 ± 0.76 ^A^	35.27 ± 0.91 ^A^
Day 0 RBC (×10^6^cell/µL)	5.83 ± 0.19 ^A^	5.90 ± 0.13 ^A^	5.53 ± 0.28 ^A^
Day 0 MCV (f/L)	61.36 ± 2.36 ^A^	62.00 ± 2.50 ^A^	63.73 ± 1.63 ^A^
Day 0 MCHC (%)	37.38 ± 0.87 ^A^	35.65 ± 0.44 ^A^	35.87 ± 1.18 ^A^
Day 0 WBC (×10^3^cell/µL)	11.66 ± 0.73 ^A^	11.80 ± 1.01 ^A^	12.23 ± 0.85 ^A^
Day 0 PLT (×10^3^cell/µL)	276.33 ± 14.49 ^A^	238.3 ± 16.017 ^A^	262.22 ± 17.43 ^A^
Day 21 HGB (g/dL)	7.15 ± 0.45 ^B^	10.83 ± 0.56 ^B^***	11.68 ± 0.47 ^A^***
Day 21 HCT (%)	23.17 ± 1.14 ^B^	29.56 ± 1.31 ^B^**	31.36 ± 1.73 ^A^**
Day 21 RBC (×10^6^cell/µL)	4.14 ± 0.15 ^B^	5.12 ± 0.11 ^B^*	5.03 ± 0.10 ^A^*
Day 21 MCV (f/L)	55.86 ± 1.00 ^A^	58.02 ± 3.17 ^A^	62.40 ± 3.73 ^A^
Day 21 MCHC (%)	30.82 ± 0.48 ^B^	36.61 ± 0.28 ^A^***	37.30 ± 0.61 ^A^***
Day 21 WBC (×10^3^ cell/µL)	31.47 ± 2.20 ^B^	25.82 ± 1.97 ^B^	16.57 ± 1.68 ^A^**^#^
Day 21 PLT (×10^3^cell/µL)	320.0 ± 11.54 ^B^	384 ± 10.21 ^B^*	390 ± 12.58 ^B^*

s/c – administration at 25 ppm once a week for two weeks by subcutaneous injection; T – administration at 25 ppm daily for two weeks topically;

*/# – significant difference from the untreated group (*) or between the treated groups (#) at the same time point (P-value ≤ 0.05);

**/*** – significant difference between groups at the same time-point at P-value ≤ 0.01/≤ 0.001;

A and B – superscript uppercase letters indicate significant differences between the day-0 and day-21 values in the same group (P-value ≤ 0.05). Values are expressed as mean ± SEM

### Biochemical assay

No significant differences between the groups’ blood biochemical parameters were noted at the same time interval. Comparing the day 0 parameter with the day 21 parameter within groups revealed no differences except for in the group that received CS/Zn NPs by s/c injection, where a significant elevation of AST enzyme level after 21 d was evident (Table 3).

**Table 3.** Effect on canine blood biochemical parameters on days 0 and 21 of chitosan-coated zinc-oxide nanoparticle (CS/ZnO NP) subcutaneous injection and topical application used in dog skin wound healing

Parameter	Untreated	CS/ZnO NP s/c	CS/ZnO NP T
Day 0 AST (U/L)	34.00 ± 3.05	35.33 ± 2.00 ^A^	37.68 ± 2.85
Day 0 ALT(U/L)	45.68 ± 4.63	44.00 ± 4.14	42.7 ± 4.26
Day 0 total proteins (g/dL)	6.34 ± 0.05	6.22 ± 0.10	6.03 ± 0.04
Day 0 ALB (g/dL)	2.08 ± 0.09	2.01 ± 0.08	2.03 ± 0.10
Day 0 urea (mg/dL)	11.41 ± 0.98	11.57 ± 0.69	12.22 ± 1.00
Day 0 CR (mg/dL)	0.96 ± 0.08	0.95 ± 0.04	1.01 ± 0.02
Day 21 AST (U/L)	36.67 ± 2.60	39.33 ± 2.33 ^B^	38.68 ± 2.40
Day 21 ALT(U/L)	46.33 ± 3.48	45.67 ± 4.05	45.00 ± 4.04
Day 21 total proteins (g/dL)	5.61 ± 0.33	6.04 ± 0.22	5.98 ± 0.21
Day 21 ALB (g/dL)	2.12 ± 0.07	2.04 ± 0.05	2.11 ± 0.17
Day 21 urea (mg/dL)	11.30 ± 0.79	11.56 ± 0.37	12.36 ± 0.80
Day 21 CR (mg/dL)	1.01 ± 0.07	1.09 ± 0.06	0.99 ± 0.47

s/c – administration at 25 ppm once a week for two weeks by subcutaneous injection; T – administration at 25 ppm daily for two weeks topically;

A and B – superscript uppercase letters indicate significant differences between the day-0 and day-21 values in the same group (P-value ≤ 0.05). Values are expressed as mean ± SEM

**Table 4.** Effect on some oxidative stress parameters in dogs on days 0 and 21 of chitosan-coated zinc-oxide nanoparticle (CS/ZnO NP) subcutaneous injection and topical application used in dog skin wound healing

Parameters	Untreated	CS/ZnO NP s/c	CS/ZnO NP T
Day 0 MDA (nmol/mL)	30.26 ± 2.36 ^A^	33.90 ± 3.11 ^A^	31.23 ± 3.15 ^A^
Day 0 CAT (U/L)	197.89 ± 15.72 ^A^	196.30 ± 15.68 ^A^	189.97 ± 14.76 ^A^
Day 0 TAC (mmol/L)	1.96 ± 0.04 ^A^	1.95 ± 0.04 ^A^	1.89 ± 0.06 ^A^
Day 21 MDA (nmol/mL)	55.77 ± 2.75 ^B^	34.23 ± 2.15 ^A^**	27.66 ± 2.33 ^A^***
Day 21 CAT (U/L)	167.96 ± 11.59 ^B^	251.67 ± 15.71 ^B^**	268.41 ± 17.31 ^B^**
Day 21 TAC (mmol/L)	0.77 ± 0.06 ^B^	1.55 ± 0.12 ^B^**	1.70 ± 0.90 ^A^***

s/c – administration at 25 ppm once a week for two weeks by subcutaneous injection; T – administration at 25 ppm daily for two weeks topically;

*/**/*** – significant difference from the untreated group at the same time point at P-value ≤ 0.05/≤ 0.01/≤ 0.001;

A and B – superscript uppercase letters indicate significant differences between the day-0 and day-21 values in the same group (P-value ≤ 0.05). Values are expressed as mean ± SEM

### Oxidative stress evaluations

At 0 d there was no significant difference in serum TAC, MDA and CAT between groups. However, at 21 d, the group that was left untreated showed a significant elevation of serum MDA from its value at the experiment’s start and significant reductions in serum TAC and CAT enzymatic activity from their levels at 0 d. Furthermore, the groups that received either topical application or injection of CS/Zn NPs had significantly higher TAC and CAT activity than the untreated group after 21 d. Interestingly, the group that received topical treatment showed higher TAC than the group that received injections.

### Transcript levels of *TNFα* and *VEGFA*

Both CS/ZnO NP injection and topical application raised the *TNFα* and *VEGFA* gene levels at all time points compared with the levels in the control untreated group (Fig. 3).

**Fig. 3.**
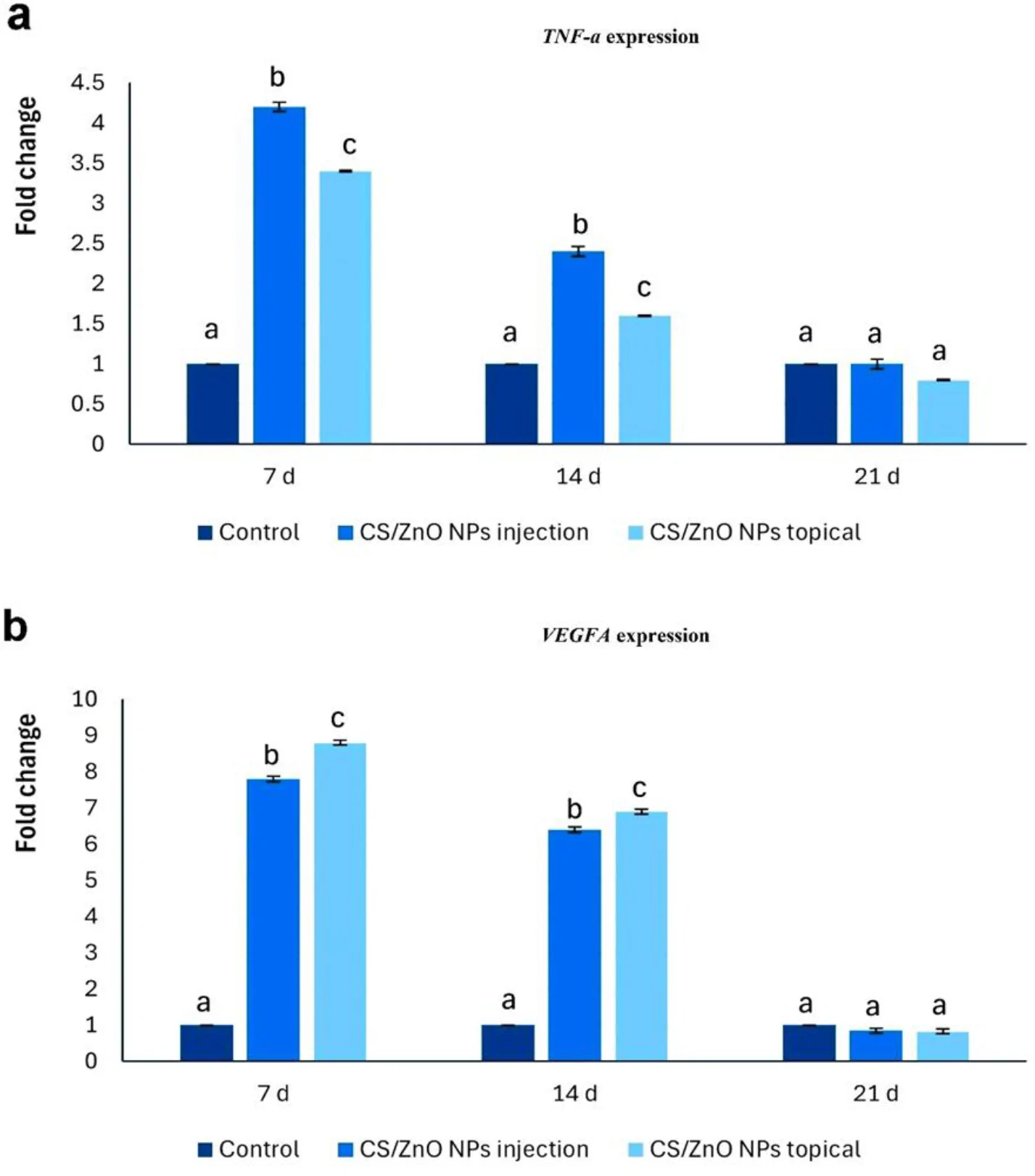
Transcript levels of a – *TNFα* and b – *VEGFA* genes on the 7^th^, 14^th^ and 21^st^ days after induction of a wound in dog skin and applying or not applying treatment. injection – administration of chitosan-coated zinc-oxide nanoparticles (CS/ZnO NPs) at 25 ppm once a week for two weeks by subcutaneous injection; topical – administration of chitosan-coated zinc-oxide nanoparticles at 25 ppm daily for two weeks topically; ^a, b and c^ – different superscript letters above columns indicate significant difference between groups at the same time point (P-value ≤ 0.05). Values are expressed as mean ± SEM

### Histopathological examination

The control untreated group showed complete epidermal ulceration with severe inflammatory cell infiltration, congestion, haemorrhage and necrosis in wound gaps on the 7^th^ day PT. On the 14^th^ day PT, the wound surfaces were covered with crust along with inflammatory cell infiltration and fibroblast proliferation. On the 21^st^ day, the wound areas showed complete epithelial regeneration with less inflammatory cell infiltration, neovascularisation, and mild collagen production (Fig. 4).

**Fig. 4.**
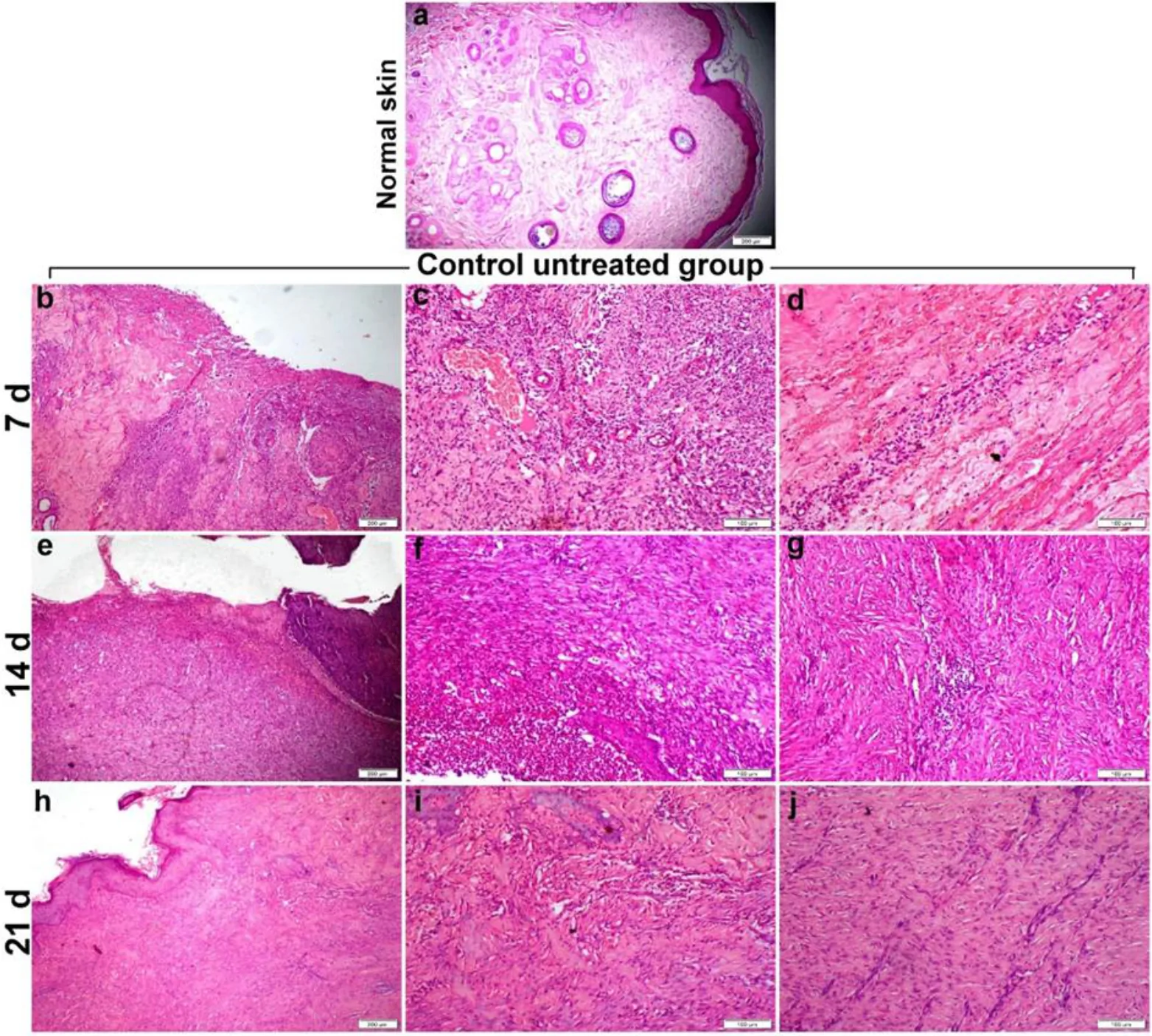
Photomicrographs of HE-stained dog skin wound sections obtained from the control untreated group. a – normal histology of intact skin; b–d – wound area on the 7^th^ day post treatment (PT) showing severe inflammation, necrosis, congestion and haemorrhage; e–g – wound area on the 14^th^ day PT showing delayed regeneration, angiogenesis and granulation tissue formation; h–j – wound area on the 21^st^ day PT showing complete re-epithelialisation, thick granulation tissue and collagen deposition

The wound sections of the CS/ZnO NP-injected group also showed heavy inflammatory cell infiltration; however, in contrast, the sections of this group had fibroblast proliferation and pronounced neovascularisation on the 7^th^ day PT. On the 14^th^ day PT, there were incomplete reepithelialisation, neovascularisation and more fibrous, less cellular granulation tissue formation over the wound bed. On the 21^st^ day PT, the wound area showed complete epidermal regeneration, fibroblast maturation and collagen fibre deposition in both the upper and lower wound areas with minimum inflammatory cell infiltration (Fig. 5).

**Fig. 5.**
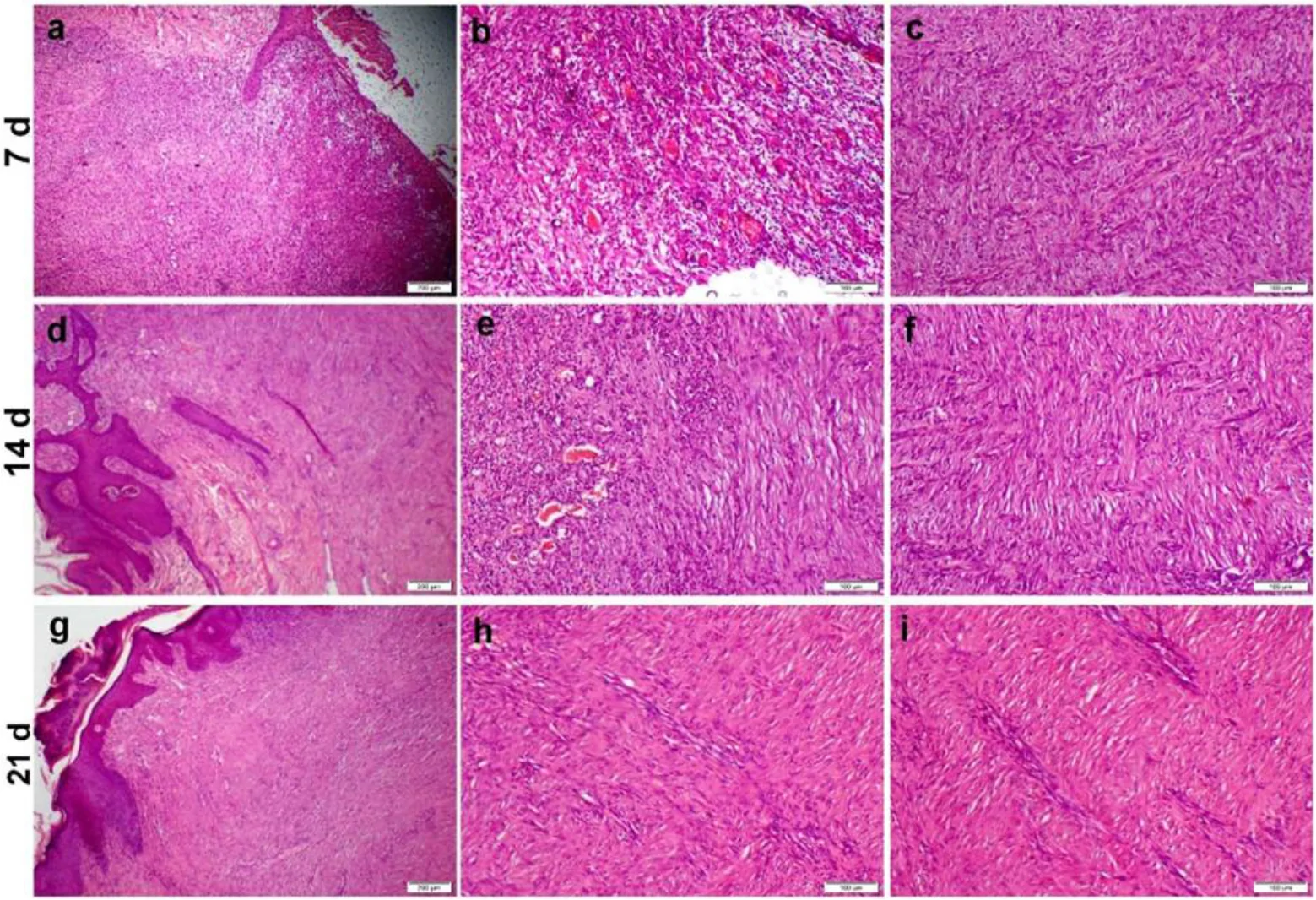
Photomicrographs of HE-stained dog skin wound sections obtained from the chitosan-coated zinc-oxide nanoparticle subcutaneously injected group treated at 25 ppm once a week for two weeks. a–c – wound area on the 7^th^ day post treatment (PT) showing severe inflammation, early onset of regeneration, angiogenesis and granulation tissue formation; d–f – wound area on the 14^th^ day PT showing complete re-epithelialisation, prominent angiogenesis, granulation tissue formation and collagen deposition; g–i – wound area on the 21^st^ day PT showing complete re-epithelialisation, granulation tissue formation and more collagen deposition

The group administered CS/ZnO NPs *via* the topical route showed profuse inflammatory cell infiltration and prominent angiogenesis in the upper layer along with extensive fibroblast proliferation and maturation in the lower layer of wounds at 7 d PT. There were complete epithelial regeneration, neovascularisation, prominent fibroblast proliferation with production of mature collagen fibre and minimum inflammatory cell infiltration at 14 d PT. At 21 d PT, the wound showed less scar tissue formation compared to other experimental groups, and the skin had begun to restore its normal histology with an intact epidermis and formation of skin appendages (Fig. 6).

**Fig. 6.**
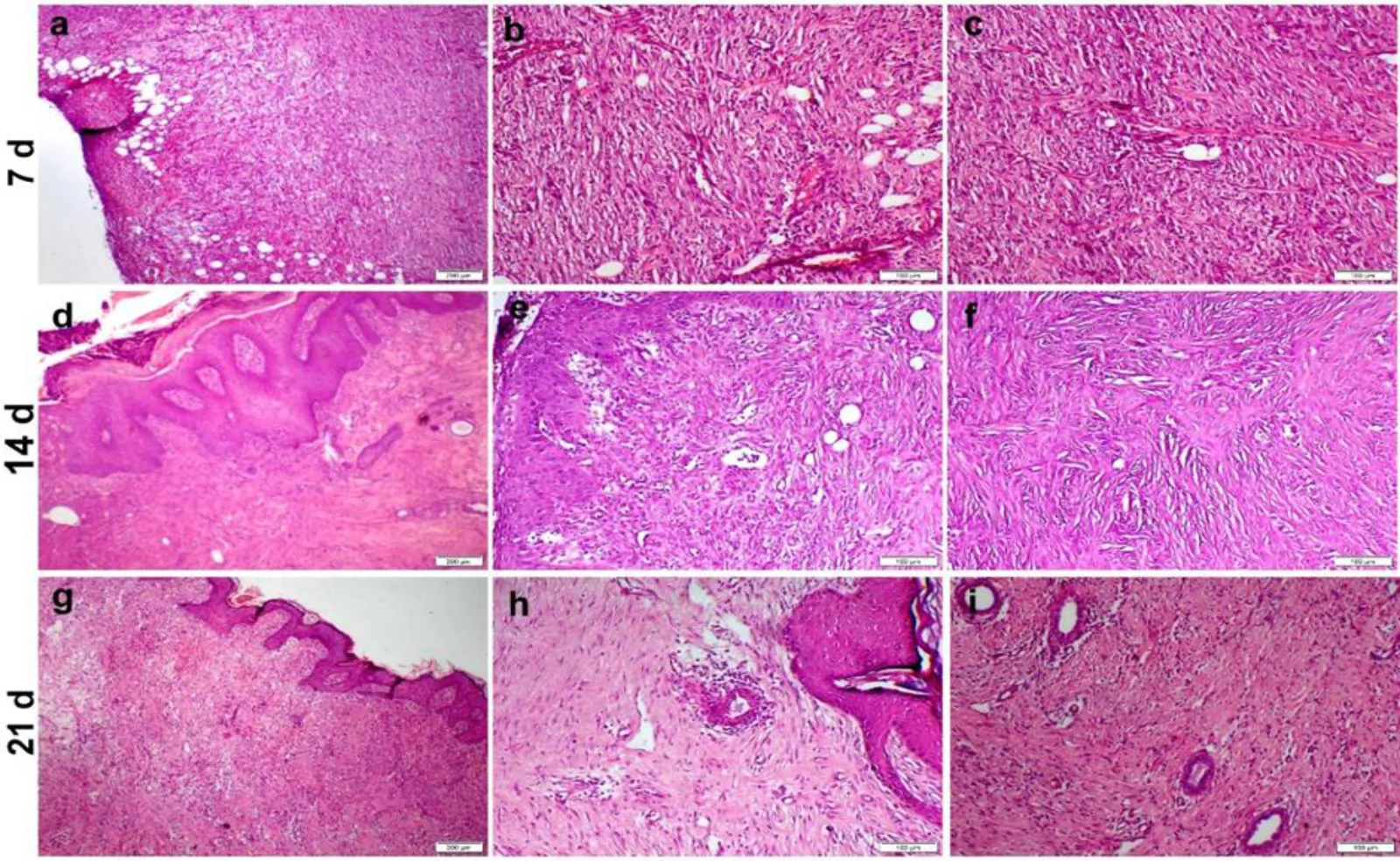
Photomicrographs of HE-stained dog skin wound sections obtained from the chitosan-coated zinc-oxide nanoparticle topically treated group having applications at 25 ppm daily for two weeks. a–c – wound area on the 7^th^ day post treatment (PT) showing early onset of angiogenesis and granulation tissue formation; d–f – wound area on the 14^th^ day PT showing complete re-epithelialisation, organised tissue formation and less scar tissue formation; g–i – wound area on the 21^st^ day PT showing less scar tissue formation and the formation of skin appendages

Masson’s trichrome stain revealed pink denatured collagen at all time points along with fine faint blue collagen haphazardly arranged in the wound bed of the control untreated group. The group treated with CS/ZnO NPs s/c had wavy bundles of mature collagen fibres distributed in the wound bed at all time points. Sections from the group administered CS/ZnO NPs *via* the topical route showed faint irregular collagen at 7 d and wavy bundles of mature collagen fibres at 14 and 21 d PT. The MTC staining intensity significantly increased in both CS/ZnO NP groups in a timedependent manner compared to its intensity in the control untreated group (Fig. 7).

**Fig. 7.**
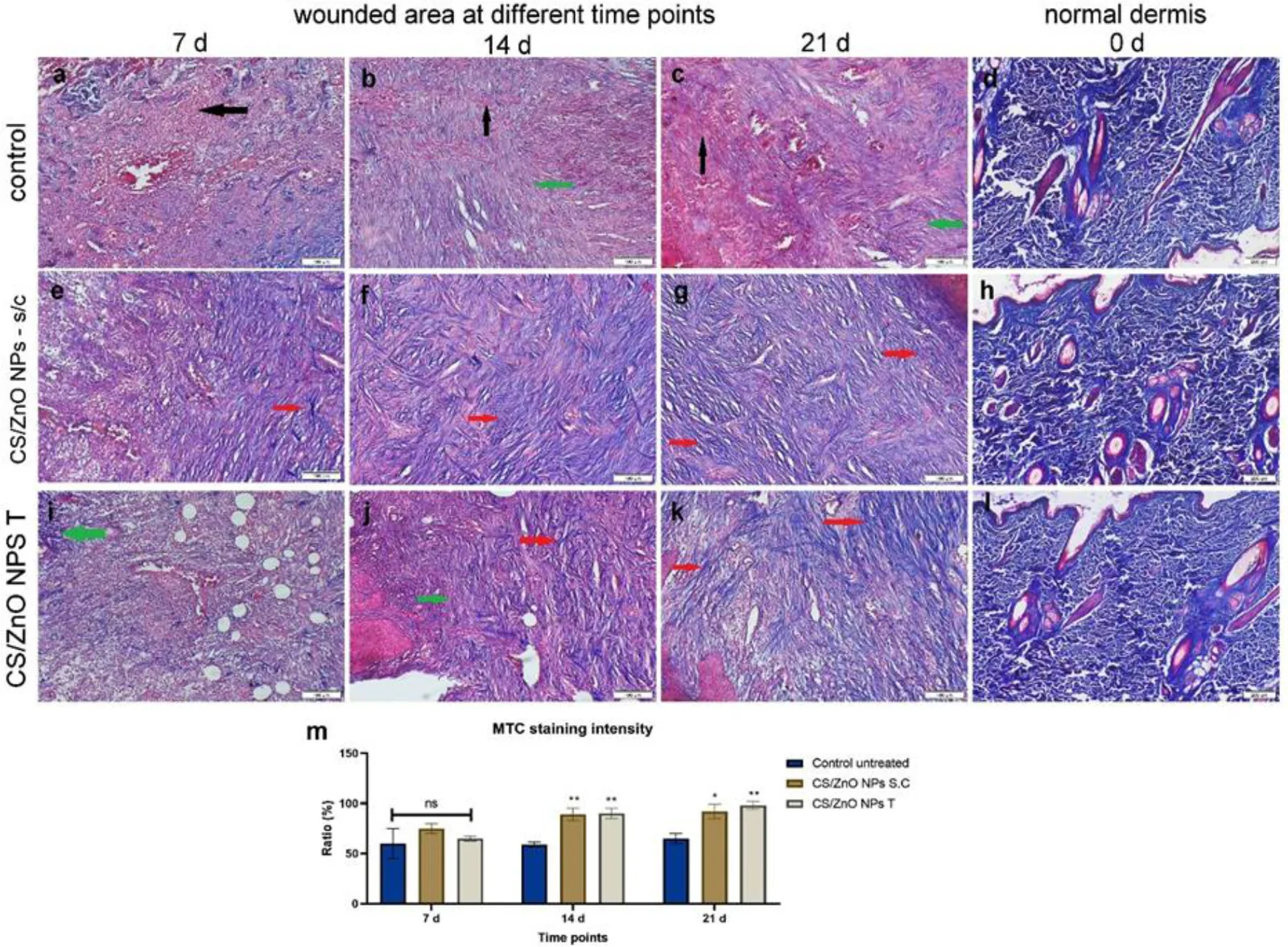
Photomicrographs of MTC-stained dog skin wound sections obtained from the control untreated group, the chitosan-coated zinc-oxide nanoparticle (CS/ZnO NP) subcutaneously injected (s/c) group treated at 25 ppm once a week for two weeks and the CS/ZnO NP topically (T) treated group having applications at 25 ppm daily for two weeks. d, h and l – normal skin on day 0 showing normal, dark-blue-stained collagen fibres arranged in wavy bundles; a–c – wound area of the control untreated group on the 7^th^, 14^th^ and 21^st^ days post treatment (PT); e–g – wound area of the CS/ZnO NPs s/c treated group on the 7^th^, 14^th^ and 21^st^ days PT; i–k – wound area of the CS/ZnO NPs T treated group on the 7^th^, 14^th^ and 21^st^ days PT. Black arrows – pink degenerated collagen fibres; green arrows – faint blue irregularly arranged collagen fibres; red arrows – dark-blue regularly arranged collagen fibres; m – Bar chart representing MTC staining intensity in each treatment group on the 7^th^, 14^th^ and 21^st^ days PT. */** – significant difference between groups at the same time point at P-value ≤ 0.05/≤ 0.01, respectively; ns – nonsignificant difference. Values are expressed as mean ± SEM

A summary histological evaluation of the wound healing processes in the treatment groups is given in Table 5

**Table 5.** Histological grading of dog skin wound healing across group treated or not treated with chitosan-coated zinc-oxide nanoparticles (CS/ZnO NPs)

	Untreated	CS/ZnO NP s/c	CS/ZnO NPs T
Day 7 re-epithelialisation	–	+	++
Day 7 inflammation	+++	++++	++++
Day 7 fibroplasia	–	++	+++
Day 7 angiogenesis	–	++	+++
Day 7 collagen deposition	–	++	+++
Day 14 re-epithelialisation	–	+++	++++
Day 14 inflammation	++++	++	++
Day 14 fibroplasia	++	++++	++++
Day 14 angiogenesis	+	++++	++++
Day 14 collagen deposition	++	++++	++++
Day 21 re-epithelialisation	++++	++++	++++
Day 21 inflammation	+	+	–
Day 21 fibroplasia	++++	+++	+
Day 21 angiogenesis	+++	+++	+
Day 21 collagen deposition	+++	++++	++

s/c – administration at 25 ppm once a week for two weeks by subcutaneous injection; T – administration at 25 ppm daily for two weeks topically; – – no changes; + – < 25% (slight changes); ++ – 25–50% (moderate changes); +++ – 50–75% (marked changes); ++++ – > 75% (extensive changes)

### Immunohistochemical staining

The control untreated group’s sections showed no expression of either VEGF or αSMA at 7 d PT and mild to moderate expression of both immune markers at 14 and 21 d. The expression of VEGF was significantly increased on the 7^th^ and 14^th^ days PT in both CS/ZnO NP-receiving groups compared with the control untreated group, but it had decreased by the 21^st^ day PT. Regarding the aSMA immunostaining, this markedly increased in both CS/ZnO NP treatment groups over the time of the experiment (Figs 8–10).

**Fig. 8.**
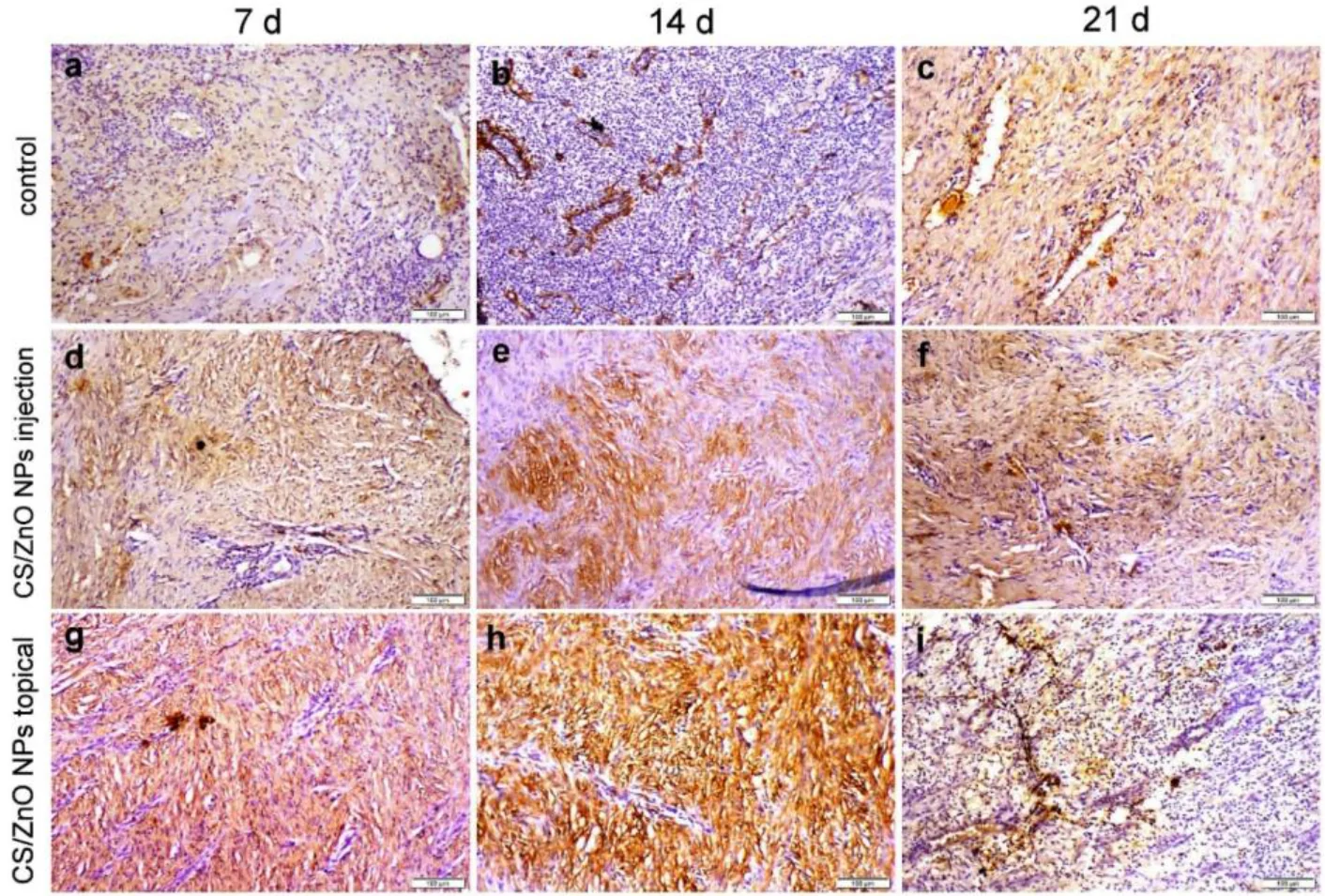
Vascular endothelial growth factor localisation in dog skin wound areas in the control untreated group, the chitosan-coated zinc-oxide nanoparticle (CS/ZnO NP) subcutaneously injected (s/c) group treated at 25 ppm once a week for two weeks and the CS/ZnO NP topically (T) treated group having applications at 25 ppm daily for two weeks. a–c – control untreated group on the 7^th^, 14^th^ and 21^st^ days post treatment (PT); d–f – CS/ZnO NP s/c treated group on the 7^th^, 14^th^ and 21^st^ days PT; g–i – CS/ZnO NP T treated group on the 7^th^, 14^th^ and 21^st^ days PT

**Fig. 9.**
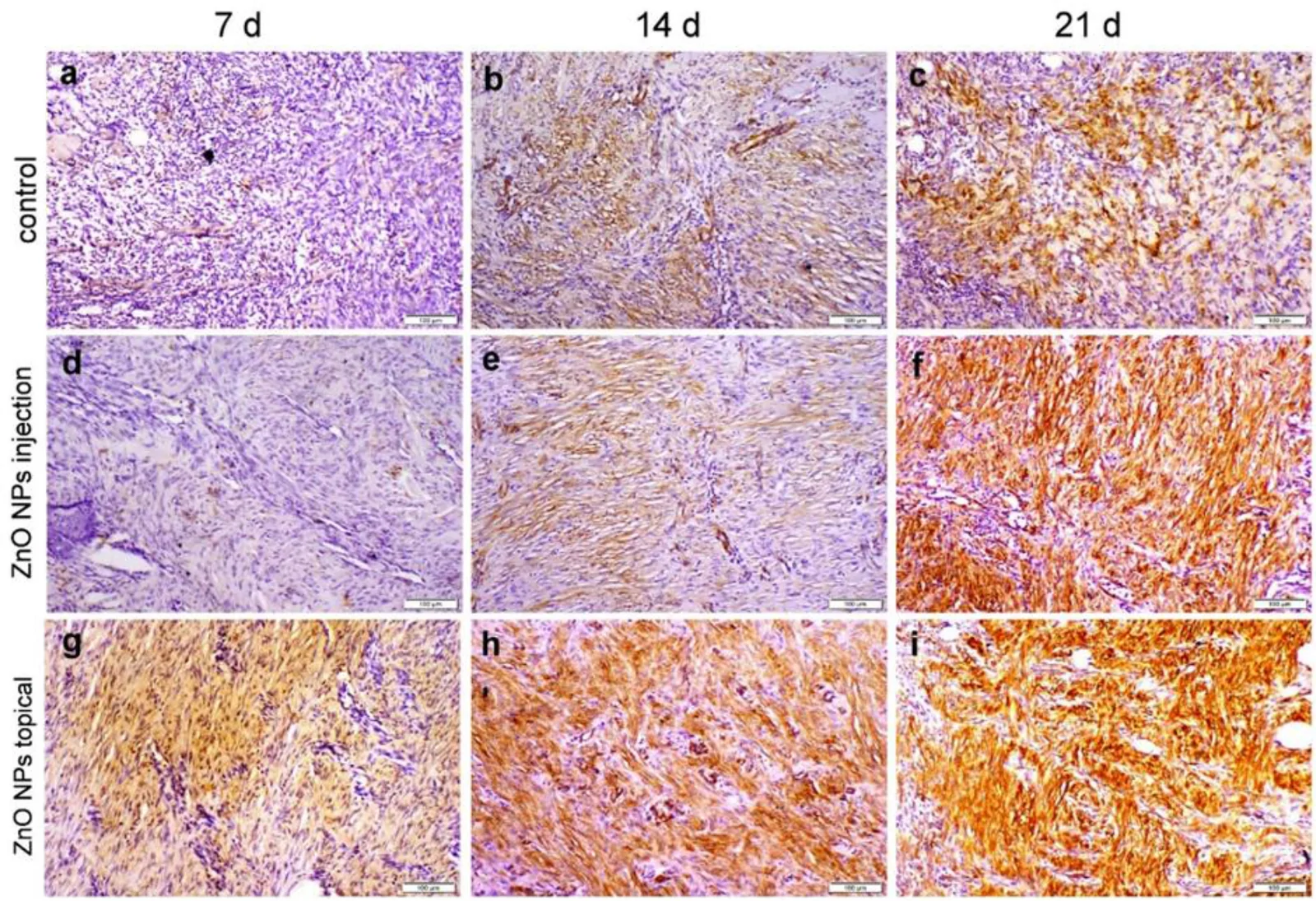
α-smooth muscle actin localisation in dog skin wound areas in the control untreated group, the chitosan-coated zinc-oxide nanoparticle (CS/ZnO NP) subcutaneously injected (s/c) group treated at 25 ppm once a week for two weeks and the CS/ZnO NP topically (T) treated group having applications at 25 ppm daily for two weeks. a–c – control untreated group on the 7^th^, 14^th^ and 21^st^ days post treatment (PT); d–f – CS/ZnO NP s/c treated group on the 7^th^, 14^th^ and 21^st^ days PT; g–i – CS/ZnO NP T treated group on the 7^th^, 14^th^ and 21^st^ days PT

**Fig. 10.**
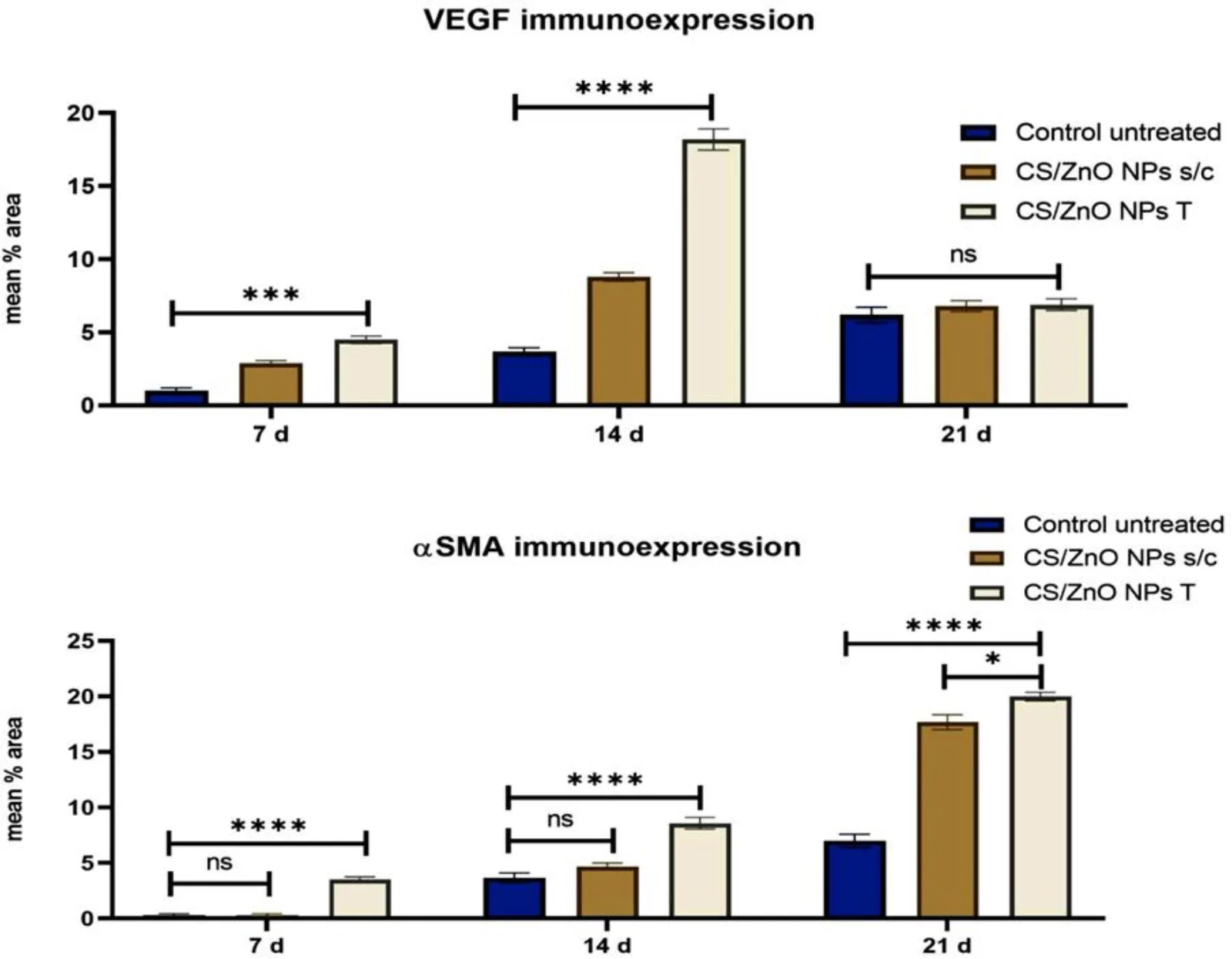
Bar chart representing the mean percentage of the tissue section area positive for vascular endothelial growth factor (VEGF) and α-smooth muscle actin (aSMA) immunoexpression in the control untreated group, the chitosan-coated zinc-oxide nanoparticle (CS/ZnO NP) subcutaneously injected (s/c) group treated at 25 ppm once a week for two weeks and the CS/ZnO NP topically (T) treated group having applications at 25 ppm daily for two weeks. Each group’s data are given on the 7^th^, 14^th^ and 21^st^ days post treatment. */***/**** – significant difference between groups at the same time point (P-value ≤ 0.05/≤ 0.001/≤ 0.0001. Values are expressed as mean ± S

## Discussion

Researchers throughout the world are becoming more interested in polymer-coated metal-oxide nanoparticles, particularly in the pharmaceutical sector (12). According to several *in vitro* and *in vivo* investigations, coating metal-oxide nanoparticles with chitosan provide numerous benefits from their changed surfaces, such as increased physicochemical stability, bioavailability and efficacy, as well as regulated release and cellular uptake of the nanoparticles, decreased toxicity and improved biological activities (1, 13). Considering recent research showing that administering 100- and 50-ppm ZnO NPs s/c to dogs can harm certain internal organs (11), the concentration of 25 ppm selected for the current study was prudent and showed no toxic side effects on the internal organs while accelerating wound healing in this species.

In the present study, control dogs received both saline and blank hydrogel as a true negative control. This design isolated the hydrogel carrier’s effect from that of CS/ZnO NPs, minimising animal usage consistently with the Three Rs principle and eliminating individual subject variability as far as possible as recommended in wound healing models. The results demonstrated that the application of CS/ZnO NPs to surgical wounds markedly accelerated healing by regulating the cellular, biochemical and molecular processes involved. Additionally, they proved that the topical application of CS/ZnO NPs to the wound area significantly enhanced healing processes at both cellular and molecular levels in comparison to s/c injection. Ramachandran *et al*. (28) found that zinc-oxide nanoparticles made with β-chitosan greatly improved wound healing in zebrafish, leading to better epidermal growth and less inflammation than was noted in the healing of the control group fish (28). Moreover, a study by Abbaszadeh *et al*. (2) showed that CS/ZnO NP dressings brought about much better healing in rats with methicillin-resistant *Staphylococcus aureus*–infected wounds, highlighting the potential of this approach for tackling difficult infections.

The data presented in the current study revealed a significant reduction in RBC count, HGB, HCT and MCHC at 21 d PT in both the control untreated and CS/ZnO NPs s/c groups, indicating the occurrence of hypochromic anaemia. Skin-wound induction can potentially lead to anaemia, but it depends on several factors. If a wound is deep or extensive, it can result in significant blood loss, which may lead to anaemia. Additionally, wounds can trigger an inflammatory response, which can affect the production of RBCs and iron metabolism (24). Interestingly, the CS/ZnO NP T group had a better haemogram than the CS/ZnO NPs s/c group, indicating the safety measures of topical application of these NPs. Platelets aid in reducing blood loss at vascular damage sites by promoting thrombin generation and fibrin formation. Furthermore, platelets express and release chemicals which promote tissue healing and influence angiogenesis, inflammation and immunological response (15). This study revealed a significant elevation of PLT in all groups after 21 d. However, the groups receiving CS/ZnO NPs had higher PLT than the control untreated group. This elevation is a prerequisite for proper wound healing (15). A significant rise in WBC count was recorded in all groups after 21 d, but it was lower in the CS/ZnO NP T group than the other two groups. Acute inflammation is a critical first step in the wound-healing process, leading to structural and functional regeneration of wounded tissue. The inflammatory cascade is initiated promptly by activated blood monocytes and tissue macrophages near the wound site, as is the release of inflammatory mediators, which cause systemic alterations such as an elevation in WBC count (14). Here, there was a significant elevation of MDA with a reduction in TAC and CAT enzyme activity after 21 d in the control untreated group. Conversely, at this time point in both CS/ZnO NP groups significantly more serum CAT and TAC activity was noted, along with less serum MDA. The CS/ZnO NP T group had more favourable results than the CS/ZnO NPs s/c group, which supports the potent antioxidant potential and free-radical scavenging properties of zinc-oxide nanoparticles as well as their beneficial effect on the overall redox status of the dog (12).

The histopathological findings revealed that application of CS/ZnO NPs to an excision wound significantly increased the recruitment of inflammatory cells, fibroblast proliferation and angiogenesis on the 7^th^ day, as well as increased the fibroblast maturation, angiogenesis and collagen fibre density and caused the deposition of fewer inflammatory cells on the 14^th^ day. Moreover, the thickness of granulation tissue was markedly decreased, reaching its lowest value on the 21^st^ day. At that point, the skin's appendages, such as hair follicles and glands, started to grow and the typical epithelialisation began to occur with deposition of both fibrous and vascular tissue. Additionally, the epithelial cells started to regenerate earlier than in the control untreated group on the 7^th^ day and had completely formed by the 14^th^ day in both CS/ZnO NP-treated groups. All these processes were better in the CS/ZnO NPs T group than the s/c injected group. We believe that the controlled release of zinc ions from the CS/ZnO NPs may help improve the healing processes, as zinc is an important element for many enzymes that aid in tissue repair (20). Several studies have proved the potential of ZnO NPs, either free or coated, for wound-healing acceleration in several animal models. Manuja *et al*. (21) determined the possibility of acacia/alginate-coated ZnO NPs to accelerate wound healing in rabbits by reducing inflammation and increasing fibroblast proliferation and maturation. A recent study in rats showed that the topical application of ZnO NPs to a wound area could raise the wound-contraction percentage and shorten the healing time (20). According to Saremi *et al*. (30), ZnO NPs sped up collagen production and wound contraction with minimum scar tissue formation. They also improved debris clearance, platelet activation, angiogenesis and collagen synthesis, and hastened re-epithelialisation (30).

The histopathological findings were confirmed by immunohistochemical staining that showed stronger VEGF and αSMA immunoexpression at various stages of wound healing in both treatment groups compared with the control untreated group. It is well established that healing processes are controlled by various cytokines and growth factors, but mainly by VEGF (7). There are several types of cells contributing to wound healing and producing VEGF, such as endothelial cells, fibroblasts, smooth muscle cells, platelets, neutrophils and macrophages (17). Vascular endothelial growth factor affects several elements of the wound-healing cascade, such as angiogenesis, collagen deposition and re-epithelialisation. The phases of angiogenesis in wound healing include vasodilation, basement membrane breakdown, endothelial cells migration and proliferation (27). Granulation tissue, a fibrovascular tissue made up of blood vessels, collagen and fibroblasts, is a crucial component of normal wound healing. Neovascularisation or angiogenesis is essential, especially at the early stage of healing processes, as it serves as a pathway for elimination of metabolites and delivery of nutrients to a wound area. In the late stage of wound healing, fibroblasts converted into myofibroblasts that express α-SMA contribute to the repair process by secreting many extracellular matrix proteins that cause the wound to contract (33).

The present study revealed that the application of CS ZnO NPs either s/c or topically boosted the mRNA levels of the *TNFα* and *VEGFA* genes at all time points. Numerous studies have focused extensively on the relationship between angiogenesis, ZnO NPs, TNF-α and VEGFA. It is reported that ZnO NPs increased VEGFA, which in turn induced angiogenesis and wound healing processes (11). In another study, modified ZnO NPs improved wound angiogenesis by raising VEGFA and lowering TNF-α (29). Shahmoradi *et al*. (32) demonstrated that the application of both ZnO NPs and CS to MRSA-infected wounds could increase VEGFA and TNF-α until the 21^st^ day PT and that it significantly reduced the wound size. We thought that by inducing inflammation, which in turn raised VEGFA expression, CS ZnO NPs enhanced angiogenesis at the early stages of wound healing. Neutrophils and macrophages were the primary inflammatory cells seen in both NP-treated groups in the current investigation. Both cells release TNF-α, which may cause keratinocytes and fibroblasts to express VEGF. The growth factor may in turn promote monocyte recruitment, because monocytes express VEGF receptors and react chemotactically to VEGF (17). Our histopathological results revealed that CS/ZnO NP initiated inflammation, angiogenesis and re-epithelialisation earlier and reduced inflammation by the 14^th^ and 21^st^ days PT, although the transcriptase levels of the *TNFα* gene remained elevated until the 21^st^ day PT. There is conflicting evidence about the role of TNF-α in wound healing. It is reported that it stimulated angiogenesis, granulation tissue formation and re-epithelialisation, which are crucial steps in accelerating wound healing (36). However, another study demonstrated that TNF-α treatment reduced both collagen deposition and granulation tissue formation (4). Furthermore, it is unclear whether this is an indirect effect resulting from a favourable or adverse impact on cells like macrophages or a direct effect on cells engaged in wound healing. The newly formed CS/ZnO NPs demonstrated not only beneficial effects but also high safety, as haematological profiles and organ function remained unchanged after 21 d of subcutaneous or topical application.

While the results from early studies are promising, more research is needed to apply chitosan-coated zincoxide nanocomposites in real-world treatments. The small sample size used in this study may have limited the results’ statistical strength and generalisability. Additionally, longer-term toxicity observations are required to fully assess systemic safety, nanoparticle clearance and potential cumulative effects. Species differences also pose a challenge, as findings in dogs may not directly translate to other animals or humans. Factors like the best formula, dosage and possible longterm effects need further study. Also, creating standardised production methods and quality controls will be essential for ensuring these nanocomposites are safe and reliable for wound-healing treatments. Moreover, because TNF-α expression remained elevated during the late phase of healing despite negligible inflammatory responses, further studies are required to elucidate its role beyond the initiation of inflammation.

## Conclusion

The findings of the present study show that both topical application and subcutaneous injection of CS/ZnO NPs accelerated wound healing by improving several cellular processes involved in healing, such as inflammation, myofibroblast migration and proliferation, neovascularisation, collagen deposition and production of the extracellular matrix. Both VEGF and a-SMA play an essential role in the inflammation, angiogenesis and contraction phases of wound repair mechanisms. Additionally, the topical application of CS/ZnO NPs was not associated with adverse changes in haematological parameters, liver and kidney biomarkers or oxidative stress markers; however, the subcutaneous application appeared to be detrimental to haematological status.

## References

[1] Abbas A., Muhammad S.A., Ashar A., Mehfooz S.A., Rauf A., Bakhsh M., Nadeem T., Fu H. (2023). Comparison of the effect of zinc oxide nanoparticles and extract of Acorus calamus applied topically on surgical wounds inflicted on the skin of rabbits. Pol J Vet Sci.

[2] Abbaszadeh A., Hosseini H., Rajabzadeh A., Zarei L. (2023). Histopathological evaluation of the application of zinc nanoparticles in conjunction with chitosan dressing on the wound healing of methicillin-resistant Staphylococcus aureus infected skin wounds in rats. Crescent J Med Biol Sci.

[3] Adel Alawadi H., Andarzbakhsh K., Rastegari A., Mohammadi Z., Aghsami M., Saadatpour F. (2024). Chitosan-Aloe vera composition loaded with zinc oxide nanoparticles for wound healing: in vitro and in vivo evaluations.

[4] Ashcroft G.S., Jeong M., Ashworth J.J., Hardman M., Jin W., Moutsopoulos N., Wild T., McCartney-Francis N., Sim D., McGrady G., Song X., Wahl S.M. (2012). Tumor necrosis factor-alpha (TNF-α) is a therapeutic target for impaired cutaneous wound healing. Wound Repair Regen.

[5] Bancroft J., Layton C., Suvarna S. (2013). Bancroft’s theory and practice of histological techniques.

[6] Borges Rosa de Moura F., Antonio Ferreira B., Helena Muniz E., Benatti Justino A., Gabriela Silva A., de Azambuja Ribeiro R.I.M., Oliveira Dantas N., Lisboa Ribeiro D., de Assis Araújo F., Salmen Espindola F., Christine Almeida Silva A., Carla Tomiosso T. (2022). Antioxidant, antiinflammatory, and wound healing effects of topical silver-doped zinc oxide and silver oxide nanocomposites. Int J Pharm.

[7] Dai T., Tanaka M., Huang Y.Y., Hamblin M.R. (2011). Chitosan preparations for wounds and burns: antimicrobial and woundhealing effects. Expert Rev Anti Infect Ther.

[8] El-Shiekh R.A., Radi M.H., Elshimy R., Abdel-Sattar E., El-Halawany A.M., Ibrahim M.A., Ali M.E., Hassanen E.I. (2025). Friedelin: a natural compound exhibited potent antibacterial, antiinflammatory, and wound healing properties against MRSA-infected wounds. Naunyn Schmiedebergs Arch Pharmacol.

[9] Elshimy R., El-Shiekh R.A., Okba M.M., Ashour R.M.S., Ibrahim M.A., Hassanen E.I., Aboul-Ella H., Ali M.E. (2025). Unveiling the antimicrobial, antivirulence, and wound-healing accelerating potentials of resveratrol against carbapenem-resistant Pseudomonas aeruginosa (CRPA)-septic wound in a murine model. Inflammopharmacology.

[10] Gurtner G.C., Werner S., Barrandon Y., Longaker M.T. (2008). Wound repair and regeneration. Nature.

[11] Harvey J.W. (2012). Introduction to veterinary hematology. Vet Hematol.

[12] Hassan A., Elebeedy D., Matar E.R., Fahmy Mohamed Elsayed A., Abd El Maksoud A.I. (2021). Investigation of angiogenesis and wound healing potential mechanisms of zinc oxide nanorods. Front Pharmacol.

[13] Hassan M.H., Emam I.A., Farghali H., Ibrahim M.A., Hassan N.H., Farroh K.Y., Hassanen E.I. (2024). Toxicological screening of zinc oxide nanoparticles in mongrel dogs after seven days of repeated subcutaneous injections. BMC Vet Res.

[14] Hassanen E.I., Khalaf A.A., Tohamy A.F., Mohammed E.R., Farroh K.Y. (2019). Toxicopathological and immunological studies on different concentrations of chitosan-coated silver nanoparticles in rats. Int J Nanomedicine.

[15] Holzheimer R.G., Steinmetz W. (2000). Local and systemic concentrations of pro-and anti-inflammatory cytokines in human wounds. Eur J Med Res.

[16] Jansen E.E., Braun A., Jansen P., Hartmann M. (2021). Platelettherapeutics to improve tissue regeneration and wound healingphysiological background and methods of preparation. Biomedicines.

[17] Karayannopoulou M., Tsioli V., Loukopoulos P., Anagnostou T.L., Giannakas N., Savvas I., Papazoglou L.G., Kaldrymidou E. (2011). Evaluation of the effectiveness of an ointment based on alkannins/shikonins on second intention wound healing in the dog. Can J Vet Res.

[18] Khalaf A., Hassanen E.I., Azouz R.A., Zaki A.R., Ibrahim M.A., Farroh K.Y., Galal M.K. (2019). Ameliorative effect of zinc oxide nanoparticles against dermal toxicity induced by lead oxide in rats. Int J Nanomedicine.

[19] Kim M.H., Jeong H.J. (2015). Zinc oxide nanoparticles suppress LPS-induced NF-ĸB activation by inducing A20, a negative regulator of NF-ĸB, in RAW 264.7 macrophages. J Nanosci Nanotechnol.

[20] Kumar S.S., Venkateswarlu P., Rao V.R., Rao G.N. (2013). Synthesis, characterization and optical properties of zinc oxide nanoparticles. Int Nano Lett.

[21] Le V.A.T., Trinh T.X., Chien P.N., Giang N.N., Zhang X.R., Nam S.Y., Heo C.Y. (2022). Evaluation of the performance of a ZnO-nanoparticle-coated hydrocolloid patch in wound healing. Polymers (Basel).

[22] Manuja A., Raguvaran R., Kumar B., Kalia A., Tripathi B.N. (2020). Accelerated healing of full thickness excised skin wound in rabbits using single application of alginate/acacia-based nanocomposites of ZnO nanoparticles. Int J Biol Macromol.

[23] Mirzaei H., Darroudi M. (2017). Zinc oxide nanoparticles: biological synthesis and biomedical applications. Ceram Int.

[24] Nemeth E., Ganz T. (2014). Anemia of inflammation. Hematol Oncol Clin North Am.

[25] Ogaly H.A., Hassan N., Elsalam R.M.A., Badawy S.E., Alsherbiny M.A., Hassan B., Al-Zahrani F.A.M., Othman G., Li C.G., Elmosalamy S.H. (2025). Cellular and molecular mechanisms of alpha lipoic acid’s protective effects against diclofenac-induced hepatorenal toxicity. J Vet Res.

[26] Percie du Sert N., Hurst V., Ahluwalia A., Alam S., Avey M.T., Baker M., Browne W.J., Clark A., Cuthill I.C., Dirnagl U., Emerson M., Garner P., Holgate S.T., Howells D.W., Karp N.A., Lazic S.E., Lidster K., MacCallum C.J., Macleod M., Pearl E.J., Petersen O.H., Rawle F., Reynolds P., Rooney K., Sena E.S., Silberberg S.D., Steckler T., Würbel H. (2020). The ARRIVE guidelines 2.0: updated guidelines for reporting animal research. PLoS Biol.

[27] Pulkkinen H.H., Kiema M., Lappalainen J.P., Toropainen A., Beter M., Tirronen A., Holappa L., Niskanen H., Kaikkonen M.U., Ylä-Herttuala S., Laakkonen J.P. (2021). BMP6/TAZ-Hippo signaling modulates angiogenesis and endothelial cell response to VEGF. Angiogenesis.

[28] Ramachandran T., Mohanraj K.G., Mary Martin T., K M.S. (2024). Enhanced wound healing with β-chitosan-zinc oxide nanoparticles: insights from zebrafish models. Cureus.

[29] Salem M., Ateya A., Shouman Z., Salama B., Hamed B., Batiha G., Ataya F., Alexiou A., Papadakis M., Abass M. (2024). Amelioration of full-thickness cutaneous wound healing using stem cell exosome and zinc oxide nanoparticles in rats. Heliyon.

[30] Saremi Z., Yari R., Khodadadi I., Tabatabaei S.M. (2016). The combined effects of nano-zinc, nano-albumin and honey in healing wounds caused by third-degree burn in male mice. J Skin Stem Cell.

[31] Shahmoradi M.K., Amini Nogorani M., Mansouri F., Zarei L. (2023). Combination of zinc nanoparticles with chitosan scaffolds increased cytokine genes on wound healing of infected rats with methicillin-resistant Staphylococcus aureus (MRSA). Adv Life Sci.

[32] Veith A.P., Henderson K., Spencer A., Sligar A.D., Baker A.B. (2019). Therapeutic strategies for enhancing angiogenesis in wound healing. Adv Drug Deliv Rev.

[33] Zabihi E., Babaei A., Shahrampour D., Arab-Bafrani Z., Mirshahidi K.S., Majidi H.J. (2019). Facile and rapid in-situ synthesis of chitosan-ZnO nano-hybrids applicable in medical purposes; a novel combination of biomineralization, ultrasound, and biosafe morphology-conducting agent. Int J Biol Macromol.

[34] Zhang J., Guan J., Niu X., Hu G., Guo S., Li Q., Xie Z., Zhang C., Wang Y. (2015). Exosomes released from human induced pluripotent stem cells-derived MSCs facilitate cutaneous wound healing by promoting collagen synthesis and angiogenesis. J Transl Med.

[35] Zhou D., Liu T., Wang S., He W., Qian W., Luo G. (2020). Effects of IL-1β and TNF-α on the expression of P311 in vascular endothelial cells and wound healing in mice. Front Physiol.

